# Dose-response relationship of egg consumption with cognitive function in rural older adults: a large-scale population-based study

**DOI:** 10.3389/fnut.2025.1566659

**Published:** 2025-05-20

**Authors:** Tingyun Ren, Yinghao Yuchi, Lange Feng, Xinlai Wang, Wei Liao, Zhenzhen Tian, Huanxiang Zhang, Yuan Tian, Yuqian Li, Chongjian Wang

**Affiliations:** ^1^Department of Epidemiology and Biostatistics, College of Public Health, Zhengzhou University, Zhengzhou, China; ^2^Department of Clinical Pharmacology, School of Pharmaceutical Science, Zhengzhou University, Zhengzhou, China; ^3^Department of Laboratory Medicine, Hongqi Hospital Affiliated to Mudanjiang Medical University, Mudanjiang, China

**Keywords:** egg consumption, mild cognitive impairment, MMSE, dose-response relationship, rural population

## Abstract

**Background:**

Previous studies had accessed the effect of egg consumption on cognitive function, but the general population-based evidence is limited and inconclusive. In addition, the optimal intake of egg remains unclear. The study aimed to investigate the association of egg consumption with mild cognitive impairment (MCI) in rural adults and then explore the recommended intake of eggs.

**Methods:**

This study included 14,550 participants from the second wave of the Henan Rural Cohort Study. Egg consumption was assessed using a validated food frequency questionnaire. Cognitive function was measured using the Mini-Mental State Examination (MMSE). MCI was defined as MMSE scores ≤ 17 for illiteracy, ≤ 20 for primary school education, or ≤ 24 for junior high school education or higher. Logistic regression analyzed the relationship between egg intake and MCI. Dose-response relationships were evaluated using restricted cubic spline (RCS) models.

**Results:**

The adjusted odds ratio (*OR*) and 95% confidence interval (*CI*) of per SD increase of daily egg consumption for MCI was 0.925 (0.891, 0.960). A U-shape dose-response relationship between egg consumption and MCI was found (*p* < 0.001), and the optimal egg intake was 87.94 g/day. The further analysis showed that *OR* (95%CI) per SD increase of egg consumption below and above the optimal intake (87.94 g/day) was 0.826 (0.774, 0.880) and 1.188 (1.056, 1.336), respectively. Sensitivity analyses showed similar results.

**Conclusion:**

Egg consumption is associated with cognitive function among rural populations and eat egg 87.94 g/day could have best benefit, supporting its potential role in dietary strategies for cognitive health.

## 1 Introduction

Mild cognitive impairment (MCI) is an early stage of cognitive disorder, characterized by noticeable deterioration in memory and thinking ability that occurs with aging and the more severe deterioration observed in dementia, with an annual progression rate to dementia of approximately 10–15% (1). With a global prevalence of 19.7% and a similar rate of 19.6% in China (2–5), MCI represents a pressing public health issue. This situation is particularly challenging in developing countries like China, where large rural populations and limited healthcare resources strain the healthcare system. These challenges highlight the importance of early intervention in MCI to delay or prevent the occurrence of dementia.

Dietary interventions have emerged as modifiable factors that may support cognitive health. Egg, a widely accessible and affordable food, are rich in nutrients such as unsaturated fatty acids (e.g., oleic and linoleic acids), antioxidants (lutein and zeaxanthin), and various B vitamins (vitamin B2, vitamin B12, folate), which have been linked to cognitive benefits (6–8). However, existing evidence on the relationship between egg consumption and cognitive function remains inconsistent. For instance, a U.S. study found that consuming more than one egg per week slowed memory decline (9), while a Chinese cohort study reported better cognitive outcomes with fewer than six eggs per week (10). In contrast, another Chinese study suggested that daily egg consumption was associated with a reduced incidence of dementia (11). Although the studies provide valuable insights into the relationship between egg intake and cognitive function, inconsistencies in the classification criteria for frequency and quantity of egg consumption across studies present the challenges for direct comparison of results to some extent.

Moreover, most existing studies have focused on urban regions with higher socioeconomic status, despite evidence showing that MCI prevalence is twice as high in rural areas (12). This disparity highlights the urgent need to explore effective cognitive health strategies tailored to rural communities. Thus, this study aimed to investigate the association between daily egg consumption and cognitive function, as well as the dose-response relationship between egg intake and cognitive outcomes, in adults aged 60 and above living in rural China.

## 2 Methods

### 2.1 Study population and design

This study population was extracted from the second wave of the Henan Rural Cohort Study, which was conducted in five rural districts of Henan Province, China (13, 14). The first wave survey of the Henan Rural Cohort Study was conducted in 2015–2017, and the second wave in 2018–2022. With the follow-up rate of 91.69%, 35,995 participants finished the second wave survey from 39,259 participants in the first wave. A total of 28,628 participants were interviewed by face-to-face surveys. For this analysis, we focused on participants aged 60 years or older (*n* = 14,960), excluding 13,668 participants younger than 60 years. An additional 410 participants with missing data on egg consumption and MMSE scores were excluded. The final analytical sample included 14,540 participants.

The Henan Province Rural Cohort Study received ethical approval from the Life Sciences Ethics Committee of Zhengzhou University and was conducted in accordance with the principles set out in the Declaration of Helsinki. All participants submitted an informed consent form before participating in the study.

### 2.2 Measurement of daily egg consumption

Daily egg consumption was assessed using a validated food frequency questionnaire (FFQ), which asked participants to report their average frequency and quantity of egg intake over the past 12 months. Participants were required to indicate how often they consumed eggs (e.g., per day, week, month, or year) and how many eggs they typically ate on each occasion. Based on this information, daily egg intake was estimated using two approaches: (1) By weight (continuous variables); The reported frequency and quantity were converted into grams per day using a standard egg weight of 62.5 grams per egg. This calculation allowed for a continuous measure of daily egg intake derived directly from the original FFQ responses.; (2) By quantity (Four Categories): Participants were further classified into four categories according to the number of eggs consumed per day: no eggs per day (0 eggs/day), between 0 and 1 egg per day (0–1 eggs/day), exactly 1 egg per day (1 egg/day), and more than 1 egg per day (>1 egg/day).

The FFQ used in this study has previously been validated within the Henan Rural Cohort and shown to have good reproducibility and reasonable validity for assessing egg consumption and other dietary components (15).

### 2.3 Definition of mild cognitive impairment

The Mini-Mental State Examination (MMSE) is a widely used and validated tool for assessing cognitive function in clinical and research settings. Originally developed by Folstein et al. (16), the MMSE evaluates multiple domains of cognition, including orientation, registration, attention, calculation, recall, and language. In this study, mild cognitive impairment (MCI) was operationally defined based on education-adjusted MMSE cut-offs, consistent with previous large-scale epidemiological studies in Chinese populations (17–21). The classification criteria were as follows: (1) Illiterate participants: MMSE scores ≤17 indicates cognitive impairment. (2) Primary school education: MMSE score ≤ 20 indicates cognitive impairment. (3) Junior high school education or higher: MMSE score ≤ 24 indicates cognitive impairment.

### 2.4 Covariates

Related covariates were selected based on their potential associations with both daily egg consumption and cognitive health outcomes, including MMSE scores and MCI status. Demographic variables included age and gender. Socioeconomic status variables included educational level, income level, and marital status. Lifestyle factors included smoking status, drinking status, high-fat diet, adequate intake of vegetables and fruits, physical activity, and body mass index (BMI). Medical history included self-reported diagnoses of hypertension, dyslipidaemia, type 2 diabetes mellitus, coronary heart disease, and stroke (22).

### 2.5 Statistical analysis

Descriptive statistics were used to summarize participant characteristics. Continuous variables were expressed as mean ± standard deviation (SD), and categorical variables were presented as frequencies and percentages. Comparisons between groups were assessed using analysis of variance (ANOVA) for continuous variables and chi-square tests for categorical variables.

Linear regression models were used to evaluate the association between daily egg consumption and MMSE scores. Beta coefficients (β), 95% confidence intervals (*CIs*), and *p*-values were reported. To assess the robustness of the model, we also conducted linear regression using square root–transformed MMSE scores. Logistic regression models were applied to analyse the association between daily egg consumption and MCI status, treating MCI as a binary outcome (MCI vs. non-MCI). Odds ratios (*ORs*) with 95% *CIs* were reported. The regression analyses included three levels of adjustment. The unadjusted model was followed by a model adjusted for age and gender. A fully adjusted model further controlled for socioeconomic status (educational level, income level, and marital status), lifestyle factors (smoking status, drinking status, high-fat diet, adequate vegetable and fruit intake, physical activity, and body mass index), and history of chronic diseases, including hypertension, dyslipidaemia, type 2 diabetes mellitus, coronary heart disease, and stroke. Restricted cubic spline (RCS) models were applied to examine potential non-linear dose-response relationships between egg intake and both MMSE score and MCI risk. Specifically, the RCS model used four knots placed at the 5th, 35th, 65th, and 95th percentiles of daily egg intake, and egg consumption = 0 g/day was set as the reference value.

To assess the robustness of the findings and explore potential effect modification, stratified analyses were performed by gender, smoking status, and drinking status. Sensitivity analyses were performed to evaluate the stability of the findings. These included: (1) excluding participants with a history of coronary heart disease or stroke, (2) using raw, non-imputed data instead of multiple imputed datasets, and (3) further adjusted medicine use of hypertension and type 2 diabetes mellitus.

Missing data rates of all covariables were less than 5%, and missing data was imputed by “mice” plot. All analyses were conducted using SPSS software version 21.0 and R software version 3.5.3. A two-tailed *p*-value < 0.05 was considered statistically significant.

## 3 Results

### 3.1 Participants characteristics

The baseline characteristics of participants, stratified by the presence of mild cognitive impairment (MCI), are summarized in Table 1. In this study, the prevalence of MCI among participants was 33.0% (4,792 out of 14,540). Compared to those without MCI, the MCI group were significantly older, more likely to be women, unmarried or not cohabiting, and had lower level of education, income, physical activity, and BMI. They were also less likely to smoke or drink alcohol, more likely to follow a low-fat diet, and had inadequate intake of vegetables and fruits. Additionally, the MCI group had higher rates of hypertension, dyslipidaemia, type 2 diabetes mellitus, coronary heart diseases, or stroke (All *p* < 0.05). Notably, participants with MCI had significantly lower egg consumption and MMSE scores (Both *p* < 0.05).

**Table 1:** Characteristic of study participants.

Variables	Normal (*n* = 9,748)	MCI (*n* = 4,792)	*P*
Age (years), mean ± SD	68.6 ± 5.2	69.8 ± 5.9	<0.001
Women, *n* (%)	5,558 (57.0)	2,909 (60.7)	<0.001
Educational level, *n* (%)			<0.001
Illiteracy	2,812 (28.8)	1,585 (33.1)	
Primary school or below	3,572 (36.6)	1,402 (29.3)	
Junior high school	2,335 (24.0)	1,482 (30.9)	
Senior high school or above	1,029 (10.6)	323 (6.7)	
Married/cohabiting, *n* (%)	8,025 (82.3)	3,753 (78.3)	<0.001
Personally average monthly income (RMB), *n* (%)			<0.001
<500	3,927 (40.3)	2,281 (47.6)	
500–	2,680 (27.5)	1,276 (26.6)	
1,000–	3,141 (32.2)	1,235 (25.8)	
Current smoking, *n* (%)	1,641 (16.8)	706 (14.7)	0.001
Current drinking, *n* (%)	1,252 (12.8)	507 (10.6)	<0.001
High-fat diet, *n* (%)	1,609 (16.5)	617 (12.9)	<0.001
Adequate vegetables and fruits intake, *n* (%)	4,414 (45.3)	1,911 (39.9)	<0.001
Physical activity, *n* (%)			<0.001
Light	3,903 (40.0)	2,256 (47.1)	
Moderate	3,723 (38.2)	1,558 (32.5)	
Vigorous	2,122 (21.8)	978 (20.4)	
Hypertension, *n* (%)	4,132 (42.4)	2,222 (46.4)	<0.001
Dyslipidemia, *n* (%)	3,389 (34.8)	1,751 (36.5)	0.035
Type 2 diabetes mellitus, *n* (%)	1,564 (16.0)	866 (18.1)	0.002
Coronary heart diseases, *n* (%)	969 (9.9)	550 (11.5)	0.004
Stroke, *n* (%)	1,441 (14.8)	981 (20.5)	<0.001
Body mass index (kg/m^2^), mean ± SD	24.6 ± 3.6	24.2 ± 3.6	<0.001
MMSE score, mean ± SD	25.1 ± 3.3	17.4 ± 4.6	<0.001
Egg consumption (g/day), mean ± SD	53.4 ± 41.5	49.5 ± 43.2	<0.001
Egg consumption group/day, *n* (%)			<0.001
0	915 (9.4)	601 (12.5)	
<1	3,066 (31.5)	1,628 (34.0)	
1	4,452 (45.7)	1,993 (41.6)	
>1	1,315 (13.5)	570 (11.9)	

### 3.2 Egg consumption and cognitive function level

Table 2 presents the relationship between egg consumption and MMSE score. After adjusting for multiple variables, daily egg intake in grams was positively associated with higher MMSE scores (β = 0.224, 95% *CI*: 0.149, 0.299 per SD increase). To address the left-skewed distribution of MMSE scores, a square root transformation was applied prior to modeling. The regression results using transformed MMSE scores remained consistent with the main analysis (Supplementary Table 1). When categorized by the number of eggs consumed per day, MMSE scores were significantly higher in participants who consumed eggs compared to those who did not. The highest scores were observed in individuals who consumed one egg per day (β = 1.021, 95% *CI*: 0.769, 1.274), followed by the group who consumed more than one egg per day (β = 0.951, 95% *CI*: 0.643, 1.258). Participants consuming less than one egg per day also showed improved MMSE scores, although the effect was smaller (β = 0.471; 95% *CI*: 0.211, 0.730).

**Table 2:** Associations of egg consumption with MMSE score [β (95% CI)].

Variables	Model 1	Model 2	Model 3
Egg consumption per SD increase	0.503 (0.418, 0.588)	0.429 (0.347, 0.512)	0.224 (0.149, 0.299)
Egg consumption group/day			
0	Ref.	Ref.	Ref.
<1	0.778 (0.476, 1.080)	0.670 (0.383, 0.958)	0.471 (0.211, 0.730)
1	1.777 (1.485, 2.069)	1.723 (1.445, 2.002)	1.021 (0.769, 1.274)
>1	1.870 (1.517, 2.222)	1.567 (1.228, 1.906)	0.951 (0.643, 1.258)
*P* for trend	<0.001	<0.001	<0.001

Model 1 was unadjusted; Model 2 was adjusted for age and gender; Model 3 was further adjusted for socioeconomic status (educational level, income level, and married status), lifestyle (smoking status, drinking status, high-fat diet, adequate vegetables and fruit intake, body mass index, and exercise), history of diseases (hypertension, dyslipidemia, type 2 diabetes mellitus, coronary heart disease, and stroke) based on Model 2.

**Figure 1:**
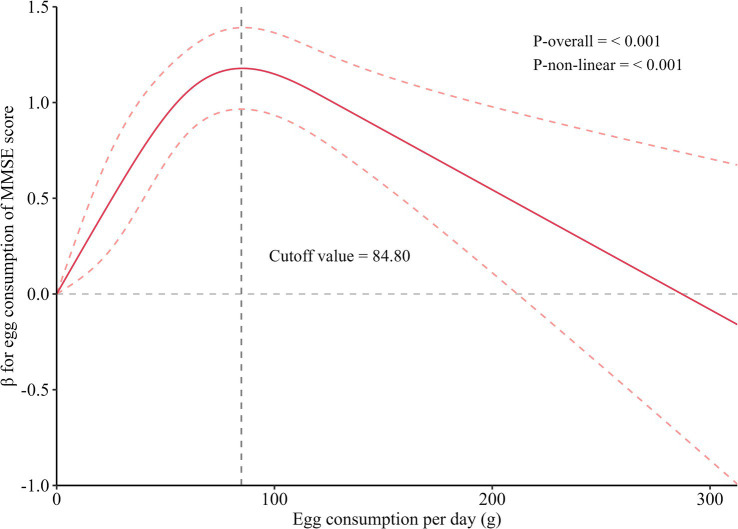
Dose-relationship of egg consumption with MMSE scores. MMSE, Mini-Mental State Examination. All estimates were adjusted for age, gender, socioeconomic status (educational level, income level, and married status), lifestyle (smoking status, drinking status, high-fat diet, adequate vegetables and fruit intake, body mass index, and exercise), history of diseases (hypertension, dyslipidemia, type 2 diabetes mellitus).

Moderate egg consumption improves cognitive function, but benefits diminish at higher intake levels. To explore the non-linear relationship between egg intake and cognitive performance, Restricted Cubic Spline (RCS) analysis was conducted. The analysis revealed a significant non-linear association, with a threshold of 84.80 g/day (Figure 1). Below this threshold, egg consumption was positively associated with MMSE scores, with β-values remaining positive (*p* < 0.05). However, beyond 84.80 g/day, β-values turned negative (*p* < 0.05), indicating that excessive egg intake may impair cognitive function (Supplementary Table 2). To account for the non-normal distribution of MMSE scores, we also performed an RCS analysis using square root–transformed MMSE as the outcome. The dose-response pattern remained similar in direction and interpretation to the main analysis using untransformed MMSE, indicating that the observed non-linear association was robust to outcome transformation (Supplementary Figure 1). It is worth noting that one egg per day in our sample corresponds to approximately 62.5 g/day, which falls within the protective range identified by the RCS model. Thus, the findings from both the categorical and continuous analyses are consistent and complementary.

### 3.3 Egg consumption and MCI

Table 3 displays that higher egg consumption was significantly associated with lower odds of MCI compared to the reference group (no egg consumption). Each standard deviation increases in daily egg intake (in grams) corresponded to a 7.5% reduction in MCI risk (*OR* = 0.925, 95% *CI*: 0.891, 0.960 per SD increase). When categorized by the number of egg consumption levels, the lowest risk was observed among participants who consumed one egg per day (*OR* = 0.700; 95% *CI*: 0.621, 0.789), followed by those consuming more than one egg per day (*OR* = 0.689; 95% *CI*: 0.594, 0.799). Even consumption of less than one egg per day was linked to a reduced risk of MCI (*OR* = 0.823; 95% *CI*: 0.728, 0.930), compared with those who did not consume eggs. A significant dose-response trend was found (*P*_trend_ < 0.001), indicating that MCI risk decreased progressively with increasing egg intake.

**Table 3:** Associations of egg consumption with MCI [OR (95% CI)].

Variables	Model 1	Model 2	Model 3
Egg consumption per SD increase	0.910 (0.878, 0.943)	0.905 (0.872, 0.938)	0.925 (0.891. 0.960)
Egg consumption group/day			
0	Ref.	Ref.	Ref.
<1	0.808 (0.718, 0.911)	0.816 (0.723, 0.920)	0.823 (0.728, 0.930)
1	0.682 (0.607, 0.765)	0.671 (0.597, 0.754)	0.700 (0.621, 0.789)
>1	0.660 (0.572, 0.761)	0.653 (0.565, 0.755)	0.689 (0.594, 0.799)
*P* for trend	<0.001	<0.001	<0.001

Model 1 was unadjusted; Model 2 was adjusted for age and gender; Model 3 was further adjusted for socioeconomic status (educational level, income level, and married status), lifestyle (smoking status, drinking status, high-fat diet, adequate vegetables and fruit intake, body mass index, and exercise), history of diseases (hypertension, dyslipidemia, type 2 diabetes mellitus, coronary heart disease, and stroke) based on Model 2.

**Figure 2:**
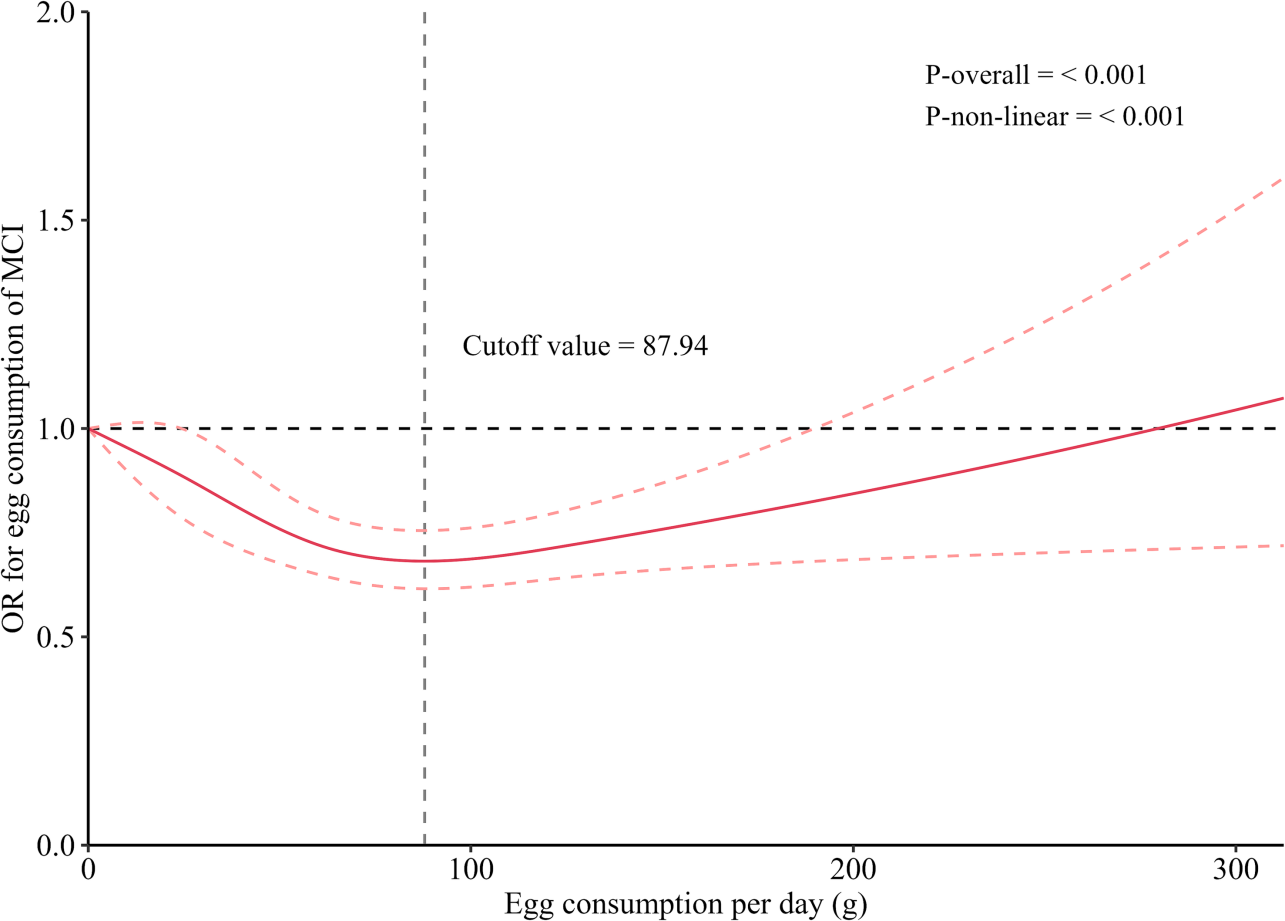
Dose-relationship of egg consumption with MCI. MCI, mild cognitive impairment. All estimates were adjusted for age, gender, socioeconomic status (educational level, income level, and married status), lifestyle (smoking status, drinking status, high-fat diet, adequate vegetables and fruit intake, body mass index, and exercise), history of diseases (hypertension, dyslipidemia, type 2 diabetes mellitus).

To further examine the dose-response relationship, RCS analysis was conducted (Figure 2). RCS results revealed a significant non-linear association between daily egg consumption and MCI risk. The the lowest odds ratio of MCI was observed at an intake of 87.94 g/day. Below this threshold, increased egg consumption was associated with a reduced risk of MCI (*p* < 0.05). However, beyond this point, the association reversed; β-values turned positive (*p* < 0.05), indicating an increased risk of MCI with excessive egg consumption. At very high intake levels, the *OR* approached 1, suggesting a diminishing or a loss of the protective effect. Notably, consuming one egg per day (~62.5 g/day) falls within the observed protective range in the spline model, aligning with the results of the categorical analysis. Thus, the two approaches consistently suggest that moderate egg intake is beneficial, while higher consumption offers no additional advantage and may even increase risk.

### 3.4 Stratified analysis and sensitivity analysis

The results of the stratified analyses, presented in Supplementary Table 3, were conducted according to gender, smoking status, and drinking status. These subgroup results were consistent with the main findings, and no significant interactions were observed. Sensitivity analyses further supported the robustness of the primary results, confirming the beneficial association between moderate daily egg consumption and improved cognitive function, as well as a reduced risk of MCI (Supplementary Tables 4, 5). Moreover, after excluding participants with a history of coronary heart disease or stroke, the association remained evident, with higher egg consumption linked to increased MMSE scores and decreased MCI risk. Similar findings were observed when analyses were repeated using non-imputed data and after additional adjustments for the use of medications related to hypertension and type 2 diabetes mellitus. These results reinforce the reliability of the observed associations and support the protective role of moderate egg consumption against cognitive decline and MCI.

## 4 Discussion

This is one of few population-based studies to demonstrate that daily egg consumption is positively associated with better cognitive function and a reduced risk of mild cognitive impairment (MCI) in a rural Chinese population, while also providing a recommended intake range. The nonlinear dose-response relationship identified through restricted cubic spline (RCS) analyses revealed thresholds of 84.80 g/day for MMSE and 87.94 g/day for MCI, above which the protective effect becomes potentially harmful.

Our findings provided a more precise recommendation for optimal egg intake to support cognitive health compared to previous research. While previous research has suggested that moderate egg consumption may slow memory loss, most lacked a clear definition of ‘moderate’ or failed to identify a threshold above which the benefits plateau or reverse. For instance, a U.S. study showed that consuming more than one egg per week was associated with slower cognitive decline, although it did not define an upper limit (9). Similarly, a recent Chinese study recommended daily egg consumption without specifying the optimal quantity (11). Conversely, another Chinese study (10) suggested that consuming fewer than six eggs per week was most effective in mitigating cognitive decline. In contrast to these findings, our study indicates that consuming one egg per day (equivalent to seven eggs per week) provides greater cognitive benefits for older adults. Furthermore, we identified 87.94 g/day as the optimal intake level, addressing a gap in the existing literature and contributing to a more detailed understanding of the relationship between egg consumption and cognitive health. Thus, these inconsistencies across studies underscore the need to investigate the underlying biological mechanisms by which egg intake influences cognitive function, to better interpret and reconcile the observed results.

The potential mechanisms underlying the protective effects of moderate egg consumption may involve several essential nutrients found in eggs, such as choline, lutein, and unsaturated fatty acids, which are known to support brain health. Lutein, an antioxidant concentrated in egg yolk, has been shown to reduce oxidative stress and inflammation in the brain, thereby enhancing cognitive performance (23–25). Additionally, unsaturated fatty acids may improve neuronal function and help lower the risk of cognitive decline (26, 27). However, excessive egg consumption may lead to elevated serum cholesterol levels, which are strongly linked to vascular burden and chronic inflammation, which can adversely affect brain health and cognitive function (28, 29). Increased cholesterol levels have also been associated with an increased risk of cardiovascular disease, which can negatively affect cerebral blood flow and lead to impaired cognitive function (30, 31). Our findings suggest that a dose-related relationship between egg consumption and cognitive benefits, demonstrating the importance of maintaining a balanced egg intake. Further research is needed to explore how individual factors such as genetic predisposition, baseline cholesterol levels and overall dietary patterns modulate the relationship between egg intake and cognitive function (32–34).

Despite these contributions, several limitations should be acknowledged. First, the cross-sectional design restricts causal inference, making it impossible to establish definitive cause-and-effect relationships between egg consumption and cognitive function. Longitudinal studies or randomized controlled trials are necessary to validate these associations. Second, reliance on self-reported dietary data may introduce recall bias and measurement error (35). Future studies should incorporate objective biomarkers, such as serum choline or phospholipid levels, to enhance the accuracy of dietary assessments. Third, residual confounding factors, such as genetic predispositions or unmeasured dietary components, cannot be entirely excluded despite adjustments for multiple covariates. Fourth, the use of the MMSE as the sole measure for assessing cognitive impairment poses limitations. Although MMSE is widely used in clinical and research settings, it has limited sensitivity for detecting mild cognitive impairment, which may result in misclassification or underestimation of MCI. Future studies should consider combining MMSE with other cognitive screening instruments or neuropsychological assessments to improve diagnostic accuracy. Finally, by focusing on a single food item, this study does not account for potential interactions between egg consumption and overall dietary patterns, which are essential for comprehensive nutritional recommendations (36). Future research should adopt a holistic approach to dietary patterns to better understand these interactions.

## 5 Conclusion

This study identified a nonlinear relationship between daily egg intake and cognitive function in a rural population. Moderate egg intake improved MMSE scores and reduced MCI risk, with optimal thresholds of approximately 85–88 g/day (equivalent to about 1.5 eggs/day). Excessive intake beyond these thresholds may pose a risk to cognitive function. These findings suggest that moderate egg consumption may support cognitive health and the early prevention of cognitive decline, emphasizing its potential role in dietary strategies for rural populations.

## Data Availability

The data analyzed in this study is subject to the following licenses/restrictions: The data that support the findings of this study are available on request from the corresponding author. Requests to access these datasets should be directed to Chongjian Wang, tjwcj2008@zzu.edu.cn.
