# Supplementary material for: Dose-response relationship of egg consumption with cognitive function in rural older adults: a large-scale population-based study
Source: Front Nutr. 2025 May 20;12:1566659. doi: 10.3389/fnut.2025.1566659 (PMC12129797; doi:10.3389/fnut.2025.1566659)
Supplement: Supplementary file 1 [file Table_1.docx]

Supplementary table list

Supplementary table 1. Associations of egg consumption with MMSE score [*β (*95% *CI)*] (suqare-root transformation)

Supplementary table 2. Association of egg consumption with MMSE and MCI stratified by optimal egg consumption

Supplementary table 3. Stratified analysis

Supplementary table 4. Sensitive analysis

Supplementary table 5. Sensitive analysis stratified by optimal egg consumption

Supplementary figure 1. Dose-relationship of egg consumption with MMSE scores (suqare-root transformation)

Supplementary table 1. Associations of egg consumption with MMSE score [*β (*95% *CI)*] (suqare-root transformation)

| **Variables** | **Model 1** | **Model 2** | **Model 3** |
| --- | --- | --- | --- |
| Egg consumption per SD increase | 0.053 (0.043, 0.063) | 0.045 (0.035, 0.055) | 0.022 (0.012, 0.031) |
| Egg consumption group /day |  |  |  |
| 0 | Ref. | Ref. | Ref. |
| <1 | 0.092 (0.056, 0.128) | 0.080 (0.045, 0.114) | 0.057 (0.026, 0.089) |
| 1 | 0.197 (0.162, 0.232) | 0.192 (0.158, 0.225) | 0.112 (0.082, 0.143) |
| >1 | 0.201 (0.159, 0.243) | 0.168 (0.127, 0.209) | 0.098 (0.060, 0.135) |
| *P* for trend | <0.001 | <0.001 | <0.001 |

Model 1 was unadjusted; Model 2 was adjusted for age and gender; Model 3 was further adjusted for socioeconomic status (educational level, income level, and married status), lifestyle (smoking status, drinking status, high-fat diet, adequate vegetables and fruit intake, body mass index, and exercise), history of diseases (hypertension, dyslipidaemia, type 2 diabetes mellitus, coronary heart disease, and stroke) based on Model 2.

Supplementary table 2. Association of egg consumption with MMSE and MCI stratified by optimal egg consumption

|  | **MMSE *β* (95% CI)** | **MCI OR (95% CI)** |
| --- | --- | --- |
| Egg consumption per day (g) |  |  |
| < 84.80 | 0.015 (0.012, 0.018) | - |
| ≥ 84.80 | -0.009 (-0.015, -0.003) | - |
| Egg consumption per day (g) |  |  |
| < 87.94 | - | 0.995 (0.994, 0.997) |
| ≥ 87.94 | - | 1.004 (1.001, 1.007) |

All estimates were adjusted for age, gender, socioeconomic status (educational level, income level, and married status), lifestyle (smoking status, drinking status, high-fat diet, adequate vegetables and fruit intake, body mass index, and exercise), history of diseases (hypertension, dyslipidemia, type 2 diabetes mellitus).

Supplementary table 3. Stratified analysis

|  | **MMSE β (95% CI)** | | ***P* for interaction** | **MCI OR (95% CI)** | | ***P* for interaction** |
| --- | --- | --- | --- | --- | --- | --- |
| **Gender** | Men | Women |  | Men | Women |  |
| Egg consumption per SD increase | 0.169 (0.071, 0.266) | 0.292 (0.179, 0.405) | 0.196 | 0.939 (0.892, 0.99) | 0.907 (0.86, 0.957) | 0.516 |
| Egg consumption group /day |  |  | 0.734 |  |  | 0.620 |
| 0 | Ref. | Ref. |  | Ref. | Ref. |  |
| <1 | 0.438 (0.015, 0.861) | 0.462 (0.132, 0.792) |  | 0.879 (0.711, 1.086) | 0.802 (0.690, 0.932) |  |
| 1 | 1.034 (0.627, 1.442) | 0.993 (0.67, 1.317) |  | 0.670 (0.546, 0.823) | 0.726 (0.626, 0.842) |  |
| >1 | 0.920 (0.470, 0.861) | 0.990 (0.553, 0.792) |  | 0.709 (0.564, 0.892) | 0.660 (0.538, 0.810) |  |
| **Current smoking** | No | Yes |  | No | Yes |  |
| Egg consumption per SD increase | 0.237 (0.151, 0.323) | 0.194 (0.044, 0.344) | 0.542 | 0.919 (0.881, 0.959) | 0.941 (0.867, 1.023) | 0.545 |
| Egg consumption group /day |  |  | 0.701 |  |  | 0.896 |
| 0 | Ref. | Ref. |  | Ref. | Ref. |  |
| <1 | 0.523 (0.240, 0.805) | 0.120 (-0.535, 0.776) |  | 0.809 (0.709, 0.922) | 0.919 (0.652, 1.296) |  |
| 1 | 1.044 (0.769, 1.320) | 0.843 (0.212, 1.474) |  | 0.701 (0.616, 0.797) | 0.690 (0.494, 0.963) |  |
| >1 | 0.979 (0.633, 0.805) | 0.796 (0.11, 0.776) |  | 0.676 (0.574, 0.797) | 0.727 (0.505, 1.046) |  |
| **Current drinking** | No | Yes |  | No | Yes |  |
| Egg consumption per SD increase | 0.235 (0.152, 0.318) | 0.143 (-0.022, 0.309) | 0.340 | 0.916 (0.879, 0.954) | 0.984 (0.893, 1.085) | 0.104 |
| Egg consumption group /day |  |  | 0.902 |  |  | 0.730 |
| 0 | Ref. | Ref. |  | Ref. | Ref. |  |
| <1 | 0.488 (0.213, 0.764) | 0.254 (-0.519, 1.027) |  | 0.816 (0.718, 0.927) | 0.905 (0.590, 1.389) |  |
| 1 | 1.021 (0.752, 1.290) | 0.918 (0.171, 1.665) |  | 0.708 (0.624, 0.802) | 0.657 (0.433, 0.997) |  |
| >1 | 0.937 (0.604, 0.764) | 0.888 (0.069, 1.027) |  | 0.678 (0.579, 0.794) | 0.755 (0.478, 1.193) |  |

Supplementary table 4. Sensitive analysis

|  | **MMSE *β* (95% CI)** | **MCI OR (95% CI)** |
| --- | --- | --- |
| Excluded participants with coronary heart diseases and stroke | |  |
| Egg consumption per SD increase | 0.222 (0.139, 0.305) | 0.916 (0.877, 0.956) |
| Egg consumption group /day |  |  |
| 0 | Ref. | Ref. |
| <1 | 0.496 (0.195, 0.796) | 0.794 (0.687, 0.919) |
| 1 | 1.040 (0.748, 1.332) | 0.695 (0.603, 0.801) |
| >1 | 1.007 (0.658, 1.356) | 0.638 (0.536, 0.759) |
| *P* for trend | <0.001 | <0.001 |
| Using the data without interpolation |  |  |
| Egg consumption per SD increase | 0.221 (0.144, 0.297) | 0.922 (0.887, 0.959) |
| Egg consumption group /day |  |  |
| 0 | Ref. | Ref. |
| <1 | 0.548 (0.283, 0.812) | 0.797 (0.702, 0.904) |
| 1 | 1.051 (0.793, 1.309) | 0.687 (0.607, 0.777) |
| >1 | 1.012 (0.698, 1.327) | 0.665 (0.570, 0.775) |
| *P* for trend | <0.001 | <0.001 |
| Further adjusted medicine use of hypertension and type 2 diabetes mellitus | | |
| Egg consumption per SD increase | 0.225 (0.150, 0.300) | 0.925 (0.891, 0.960) |
| Egg consumption group /day |  |  |
| 0 | Ref. | Ref. |
| <1 | 0.475 (0.216, 0.735) | 0.823 (0.728, 0.930) |
| 1 | 1.027 (0.774, 1.280) | 0.699 (0.621, 0.789) |
| >1 | 0.956 (0.649, 1.264) | 0.688 (0.594, 0.799) |
| *P* for trend | <0.001 | <0.001 |

All estimates were adjusted for age, gender, socioeconomic status (educational level, income level, and married status), lifestyle (smoking status, drinking status, high-fat diet, adequate vegetables and fruit intake, body mass index, and exercise), history of diseases (hypertension, dyslipidemia, type 2 diabetes mellitus).

Supplementary table 5. Sensitive analysis stratified by optimal egg consumption

|  | **MMSE *β* (95% CI)** | **MCI OR (95% CI)** |
| --- | --- | --- |
| Excluded participants with coronary heart diseases and stroke | |  |
| Egg consumption per day (g) |  |  |
| < 84.80 | 0.016 (0.012, 0.019) | - |
| ≥ 84.80 | -0.008 (-0.015, -0.002) | - |
| Egg consumption per day (g) |  |  |
| < 87.94 | - | 0.995 (0.994, 0.997) |
| ≥ 87.94 | - | 1.003 (1.000, 1.007) |
| Using the data without interpolation |  |  |
| Excluded participants with coronary heart diseases and stroke | |  |
| Egg consumption per day (g) |  |  |
| < 84.80 | 0.015 (0.011, 0.018) | - |
| ≥ 84.80 | -0.009 (-0.015, -0.003) | - |
| Egg consumption per day (g) |  |  |
| < 87.94 | - | 0.995 (0.994, 0.997) |
| ≥ 87.94 | - | 1.004 (1.001, 1.007) |
| Further adjusted medicine use of hypertension and type 2 diabetes mellitus | |  |
| Egg consumption per day (g) |  |  |
| < 84.80 | 0.015 (0.012, 0.018) | - |
| ≥ 84.80 | -0.009 (-0.015, -0.003) | - |
| Egg consumption per day (g) |  |  |
| < 87.94 | - | 0.996 (0.994, 0.997) |
| ≥ 87.94 | - | 1.004 (1.001, 1.007) |

All estimates were adjusted for age, gender, socioeconomic status (educational level, income level, and married status), lifestyle (smoking status, drinking status, high-fat diet, adequate vegetables and fruit intake, body mass index, and exercise), history of diseases (hypertension, dyslipidemia, type 2 diabetes mellitus).


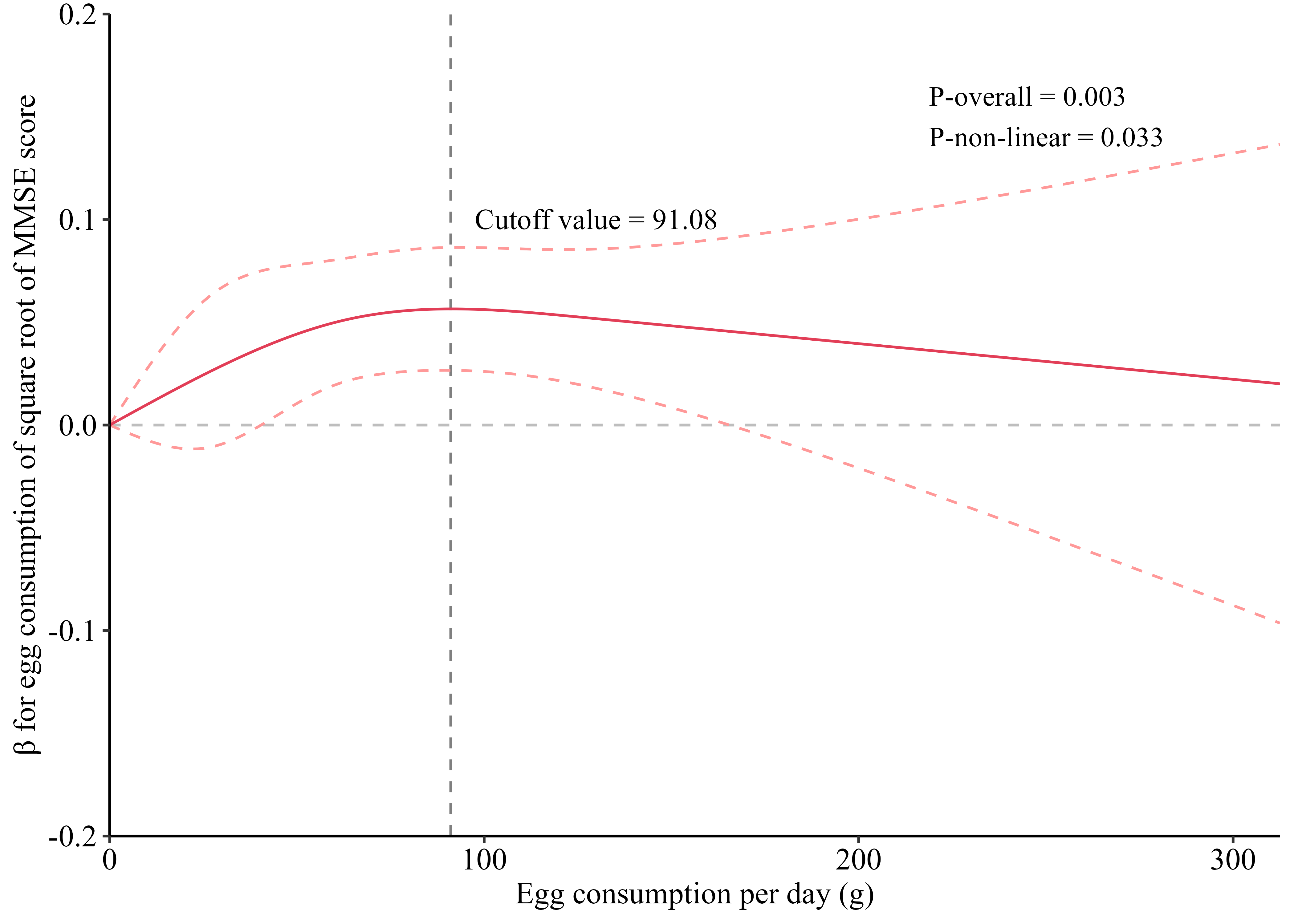


Supplementary figure 1. Dose-relationship of egg consumption with MMSE scores (suqare-root transformation)

Abbreviation: MMSE: Mini-Mental State Examination.

All estimates were adjusted for age, gender, socioeconomic status (educational level, income level, and married status), lifestyle (smoking status, drinking status, high-fat diet, adequate vegetables and fruit intake, body mass index, and exercise), history of diseases (hypertension, dyslipidaemia, type 2 diabetes mellitus)
